# From virtual practice to real-world function the transfer problem in digital neurorehabilitation

**DOI:** 10.3389/fneur.2026.1976066

**Published:** 2026-10-23

**Authors:** Mustafa Karadag, Mehmet Bahadır Ercis, Mohammad Alimoradi, Dilek Karadağ, Ceyhun Kabal, Izzet Karakulak, Mehmet Bayansalduz, Levent Ceylan, Mojtaba Iranmanesh, Mohammad Alghosi

**Affiliations:** 1 Faculty of Sports Sciences, Firat University, Elazig, Türkiye; 2 Department of Sports Management, Faculty of Sports Sciences, Atatürk University, Erzurum, Türkiye; 3 Department of Sports Injuries and Corrective Exercises, Faculty of Sports Sciences, Shahid Bahonar University of Kerman, Kerman, Iran; 4 HERC – Health, Exercise & Research Center, Dubai, United Arab Emirates; 5 Department of Coaching Education, Faculty of Sports Sciences, Eurasia University, Trabzon, Türkiye; 6 Department of Sports Management, Faculty of Sports Sciences, Mardin Artuklu University, Mardin, Türkiye; 7 Department of Sports Management, Faculty of Sport Sciences, Istanbul Topkapi University, Istanbul, Türkiye; 8 Faculty of Sport Sciences, Hitit University, Çorum, Türkiye; 9 Department of Physical Education, Technical and Vocational University (TVU), Tehran, Iran

**Keywords:** digital neurorehabilitation, ecological validity, motor learning, transfer of learning, virtual reality

## Abstract

Digital technologies have expanded opportunities for neurorehabilitation by enabling intensive, interactive, and adaptable motor practice. However, improvements achieved within virtual environments, rehabilitation games, or technology-based assessments do not necessarily translate into meaningful changes in everyday function. This Mini Review examines the transfer problem in digital neurorehabilitation and discusses key factors that may limit the generalization of training-related gains to real-world performance. We synthesize evidence regarding task specificity, sensory and perception-action differences between digital and physical environments, cognitive and contextual demands, practice variability, and patient-specific constraints that influence transfer. Based on these mechanisms, we propose a transfer-oriented framework consisting of five interconnected stages: skill acquisition, adaptive variability, cognitive-motor integration, contextualized real-world practice, and ecological evaluation. This framework emphasizes that transfer should be deliberately targeted rather than assumed as a consequence of successful digital training. We further discuss how virtual reality, augmented reality, wearable sensing, and hybrid rehabilitation models may support different stages of this process when integrated within adaptive and personalized approaches. Future digital rehabilitation research should move beyond evaluating performance within trained tasks and incorporate measures of functional and ecological transfer, including real-world mobility, activity, and participation. A shift from technology-centered rehabilitation toward transfer-oriented design may enhance the clinical impact of digital interventions and support meaningful improvements in daily life.

## Highlights

- Digital rehabilitation gains do not automatically transfer to real-world function; successful performance in virtual environments should not be assumed to represent meaningful daily-life improvements.
- Transfer is influenced by task specificity, sensory and contextual differences, cognitive-motor demands, practice variability, and individual patient characteristics.
- A transfer-oriented framework integrating adaptive practice, real-world application, and ecological evaluation may improve the clinical impact of digital neurorehabilitation.

## 1 Introduction

Neurological disorders are a major cause of long-term disability and frequently result in persistent impairments in motor function, including deficits in gait, balance, coordination, and upper-limb control (1–3). These impairments can substantially restrict independence, participation, and quality of life, making effective motor rehabilitation a central component of neurological care (4). Conventional rehabilitation is largely based on repetitive and task-specific practice, principles that promote motor learning and experience-dependent neuroplasticity (3, 5). However, the intensity, duration, and accessibility of conventional therapy are often constrained by limited clinical resources, mobility restrictions, and difficulties in sustaining long-term engagement (6). Digital technologies have expanded the possibilities for delivering motor rehabilitation. Virtual reality (VR), augmented reality (AR), exergaming, wearable sensors, and telerehabilitation platforms can provide intensive, repetitive, and interactive practice while allowing task difficulty and feedback to be adjusted according to individual performance (7, 8). These technologies may also increase engagement through gamification and provide real-time visual, auditory, or haptic feedback to support error detection and motor learning (8, 9). Across neurological populations, digital interventions have been investigated for the rehabilitation of upper-limb function, gait, balance, mobility, and activities of daily living (10–12).

Despite their growing use, digital training environments do not fully reproduce real-world conditions, and gains achieved during training may not generalize to everyday activities (13). Transfer depends partly on the correspondence between trained tasks and the sensory, motor, cognitive, and contextual demands of target activities (14, 15). For example, Mendes et al. (16) found that transfer from VR training to an untrained functional task in people with Parkinson’s disease depended on the motor and cognitive demands of the games. Everyday activities such as community mobility also require adaptation to changing environments and competing cognitive demands (17, 18). Clinical measures may capture what individuals can do under structured conditions rather than what they do in daily life (19), while wearable technologies may help assess this distinction (20).

Evidence regarding transfer varies across outcomes. One study found that VR reduces activity limitations after stroke, but did not establish improvements in free-living performance or participation (21). Thus, insufficient evidence of real-world transfer should not be interpreted as proof of transfer failure. This Mini Review examines factors that may facilitate or constrain transfer, including task specificity, sensory and contextual demands, cognitive-motor complexity, practice variability, individual characteristics, and ecological outcome assessment.

## 2 Methods

We searched PubMed, Scopus, and Web of Science from database inception to August 2026 using combinations of terms related to digital neurorehabilitation, technology-assisted rehabilitation, motor learning, transfer, and real-world function. Search terms included *digital neurorehabilitation*, *virtual reality*, *augmented reality*, *exergaming*, *telerehabilitation*, *wearable technology*, *motor learning*, *transfer*, *generalization*, *task specificity*, *dual-task*, *ecological validity*, and *real-world function*. No language restrictions were applied. Search strategies were adapted to each database. An example search string was: (“virtual reality” OR exergaming OR “digital rehabilitation”) AND (“motor learning” OR transfer OR generalization) AND (neurological OR stroke OR Parkinson*). Two reviewers screened records by title and abstract and assessed potentially relevant articles in full text. Peer-reviewed studies and reviews were eligible if they examined digital rehabilitation in neurological populations and addressed transfer or generalization beyond the trained task or controlled environment. Studies focusing exclusively on non-neurological populations or technological performance without relevant motor, functional, or real-world outcomes were excluded. Sources were selected for their relevance to the review’s main themes and synthesized narratively. Two reviewers screened records by title and abstract and assessed potentially relevant articles in full text. However, given the Mini Review format, no formal risk-of-bias assessment or meta-analysis was conducted. The narrative approach to source selection and the absence of formal quality appraisal may have introduced selection bias and limited the comprehensiveness and reproducibility of the review.

## 3 Understanding the transfer problem: from task performance to real-world function

Transfer is a fundamental concept in digital neurorehabilitation, as rehabilitation aims to enable individuals to apply acquired skills to meaningful daily activities (14). However, improvements in virtual tasks or standardized assessments do not necessarily indicate real-world functional gains. Near transfer refers to generalization to similar tasks, whereas far transfer involves tasks with substantially different motor, cognitive, or contextual demands (15, 22). In this review, transfer also encompasses functional changes in daily activities and ecological performance in real-world environments.

Digital interventions may improve trained-task performance without producing equivalent gains in daily mobility, participation, or independence. This may reflect differences between controlled digital environments and the sensory, environmental, and cognitive demands of everyday activities (22, 23). Similarly, improvements in trained dual-task activities may not generalize to untrained contexts (24). Therefore, transfer should be assessed directly rather than inferred from training gains (25). Wearable sensors and mobile technologies may support the assessment of mobility and behavior in naturalistic environments (20, 26), although their use in evaluating transfer remains limited.

Transfer should therefore be evaluated across trained tasks, untrained activities, and real-world performance to determine whether learning generalizes to meaningful everyday function.

## 4 Factors influencing transfer in digital neurorehabilitation

Digital neurorehabilitation may improve trained-task performance and functional outcomes, but the extent of generalization to real-world activities varies. Distinguishing demonstrated limited transfer from inconclusive findings due to inadequate assessment is therefore essential. This section examines individual, task-related, sensory, cognitive, and environmental factors that may constrain or facilitate transfer.

### 4.1 The specificity paradox: learning the task versus learning to adapt

Task-specific practice is a fundamental principle of neurorehabilitation, promoting motor learning through repeated, meaningful practice (27). Digital technologies can support this process by structuring repetitions, providing immediate feedback, and progressively adjusting task difficulty (9, 22). However, highly specific practice may also promote learning that is dependent on the trained task or context. This creates a specificity paradox: training must target relevant motor behaviors while avoiding excessive reliance on narrow and predictable conditions (28). Individuals may improve substantially during repeated virtual reaching, gait, or obstacle-avoidance tasks but struggle when movement, sensory, or environmental demands change.

Therefore, transfer-oriented rehabilitation should define specificity not as repeating a single movement, but as preparing individuals for the range of functional demands they will encounter. For example, community mobility training should progressively incorporate variability in speed, direction, environmental constraints, and attentional demands rather than relying solely on predictable practice.

### 4.2 The sensory and perception-action gap

Another barrier to transfer is the difference between sensory information available in digital and physical environments. Successful movement depends on integrating visual, proprioceptive, vestibular, tactile, and auditory information, which digital systems may not fully reproduce (9). These differences can alter perception–action coupling. A virtual obstacle may differ from a physical obstacle in depth cues, haptic information, physical consequences, and perceived affordances. Consequently, motor strategies developed during virtual practice may not fully transfer to similar real-world situations. Evidence of limited transfer and retention of locomotor adaptations from virtual to physical obstacle avoidance supports this concern (13).

Improving sensory fidelity through physical interaction, haptic feedback, or real-world movement constraints may help reduce this gap. However, greater technological realism does not necessarily guarantee improved transfer and requires direct evaluation.

### 4.3 The cognitive-contextual complexity

Real-world activities require continuous attention, adaptation to unexpected events, and management of multiple cognitive and environmental demands. Community ambulation, for example, may involve obstacle avoidance while monitoring traffic, conversing, or responding to distractions (29). In contrast, rehabilitation tasks are often performed under predictable and controlled conditions (23). Although this may facilitate safe task acquisition, prolonged practice in simplified environments may provide limited preparation for everyday complexity. Cognitive-motor and dual-task training can increase ecological relevance, but improvements may remain specific to the trained task (30).

Therefore, cognitive demands should be progressively varied to reflect real-world challenges. Digital technologies can support this process by manipulating distractions, task complexity, response demands, and uncertainty within a controlled and safe environment.

### 4.4 Repetition without variability

Digital rehabilitation can provide high volumes of practice, but repetition alone does not guarantee transferable learning. Highly repetitive and predictable practice may improve performance within the trained context while limiting adaptation to changing task or environmental demands (31, 32). Variable and random practice may better promote retention and transfer by exposing individuals to a broader range of movement solutions (31). Digital technologies can readily manipulate this variability by altering movement, sensory, environmental, or cognitive demands across repetitions (22, 23).

Importantly, increasing difficulty is not the same as increasing adaptability. Transfer-oriented interventions should therefore use systematic practice variability to prepare individuals for the changing demands of real-world function, rather than simply making tasks more challenging.

### 4.5 Patient-specific constraints on transfer

Transfer is influenced by individual characteristics, including motor control, sensory processing, cognition, fatigue, and adaptability (33). Differences in impairment severity and technological familiarity may also affect generalization beyond the training environment (33, 34). Consequently, digital interventions may not produce equivalent transfer across individuals, particularly when cognitive or sensory impairments and fluctuating symptoms limit adaptation (28). Environmental and social factors, including caregiver support, home accessibility, opportunities for daily activities, confidence, digital access, and adherence, may further influence real-world performance (25). Thus, limited improvements in daily activities may reflect environmental barriers or restricted opportunities rather than failed transfer.

Overall, transfer should not be assumed from successful digital training. Task specificity, sensory and contextual demands, cognitive complexity, practice variability, individual characteristics, and environmental factors may influence whether learning generalizes to real-world function. These considerations underpin the proposed framework, which progresses from controlled skill acquisition to adaptive variability, cognitive-motor integration, contextualized practice, and ecological evaluation.

## 5 Toward transfer-oriented digital neurorehabilitation

The limitations described above suggest that transfer should be an explicit therapeutic objective rather than an automatic consequence of digital training. Digital technologies can therefore support a progression from controlled skill acquisition toward increasingly variable, cognitively demanding, and ecologically relevant performance. We propose a transfer-oriented framework for digital neurorehabilitation consisting of five interconnected stages: (1) skill acquisition, (2) adaptive variability, (3) cognitive-motor integration, (4) contextualized real-world practice, and (5) ecological evaluation. These stages may overlap depending on the individual’s condition, functional capacity, and rehabilitation goals, but together provide a conceptual progression from learning a skill in a controlled environment to demonstrating its usefulness in everyday life.

### 5.1 Stage 1: skill acquisition in controlled environments

The first stage focuses on acquiring or restoring a specific motor skill under controlled conditions. Digital technologies can support high repetition, immediate feedback, individualized task difficulty, and safe practice.

Controlled environments may be particularly valuable for individuals with substantial impairments by simplifying task demands while preserving meaningful practice (35). VR, interactive systems, and exergaming can adjust movement complexity, provide augmented feedback, and support engagement (34, 36). The primary objective is successful acquisition of the target skill: *Can the individual perform the movement successfully under controlled conditions?* However, successful performance is only the starting point. Once basic skill acquisition is established, rehabilitation should progressively move beyond predictable and repetitive practice.

### 5.2 Stage 2: promoting adaptability through variability

The second stage reduces dependence on a single task configuration by systematically introducing variability. Because daily activities vary in speed, timing, spatial demands, and environmental conditions (37–39), digital systems can modify these factors across practice trials (23). Importantly, increasing variability is not simply increasing difficulty. Transfer-oriented training should expose individuals to different task and environmental constraints, encouraging adaptable movement strategies (40). The primary question is: *Can the individual adapt the learned skill when task conditions change?* This progression may be particularly important in neurological rehabilitation, where strategies effective in predictable settings may not remain effective when environmental demands change.

### 5.3 Stage 3: integrating cognitive and motor demands

As rehabilitation progresses, motor skills should increasingly be practiced under cognitive demands similar to those encountered in daily life. Everyday activities often require movement alongside attention, planning, decision-making, memory, and responses to unexpected events (30, 41).

Digital environments can progressively integrate cognitive and motor demands by introducing distractions, competing stimuli, memory tasks, decision-making, or changing environmental cues. These demands should be relevant to the functional goal rather than simply added to increase task complexity (23, 30). The primary question is: *Can the individual maintain or adapt motor performance when cognitive demands increase?* Because improvements in cognitive-motor training may remain task-specific, cognitive demands should also be varied across practice.

### 5.4 Stage 4: contextualized and real-world practice

The next stage aims to reduce the gap between digital practice and real-world function. Because even immersive virtual environments cannot fully reproduce everyday sensory and physical demands, practice should progressively incorporate real or partially real environments (22, 41). AR can support this transition by integrating digital cues with actual movement in the physical world (42), while wearable technologies can extend monitoring and feedback into home and community settings (43). The primary question is: *Can the individual apply the learned skill in the environment where it is needed?* This stage represents a critical shift from simulating function to performing function.

### 5.5 Stage 5: ecological evaluation of transfer

A transfer-oriented approach also requires evaluating outcomes beyond performance in games or standardized clinical tests. Assessment should include real-world mobility, activity, participation, and independence. Wearable sensors and mobile technologies can support ecological evaluation by monitoring behavior in naturalistic environments and distinguishing between capacity, which refers to what an individual can do under structured conditions, and performance, which reflects what they actually do in daily life (20, 23, 44). The primary question is: *Has the intervention produced a meaningful change in real-world behavior and function?* Ecological evaluation should also guide subsequent rehabilitation by identifying situations where transfer remains limited and informing further personalization of digital training.

### 5.6 From a linear pathway to an adaptive rehabilitation loop

Although the five stages provide a conceptual progression, rehabilitation is unlikely to follow a strictly linear sequence. Transfer may occur in some domains while remaining limited in others. A more useful approach is an adaptive rehabilitation loop, in which contextualized practice and ecological evaluation identify situations where performance deteriorates. These challenges can then be reintroduced into controlled or digital environments for targeted retraining. Accordingly, digital rehabilitation can be viewed as a dynamic cycle: *controlled practice → adaptive variability → cognitive-motor integration → real-world application → ecological feedback → personalized retraining.*

This approach positions technology not simply as a tool for delivering exercises, but as part of a system that supports continuous assessment, adaptation, and progression of learning across contexts. Figure 1 summarizes this transfer-oriented framework.

**Figure 1:**
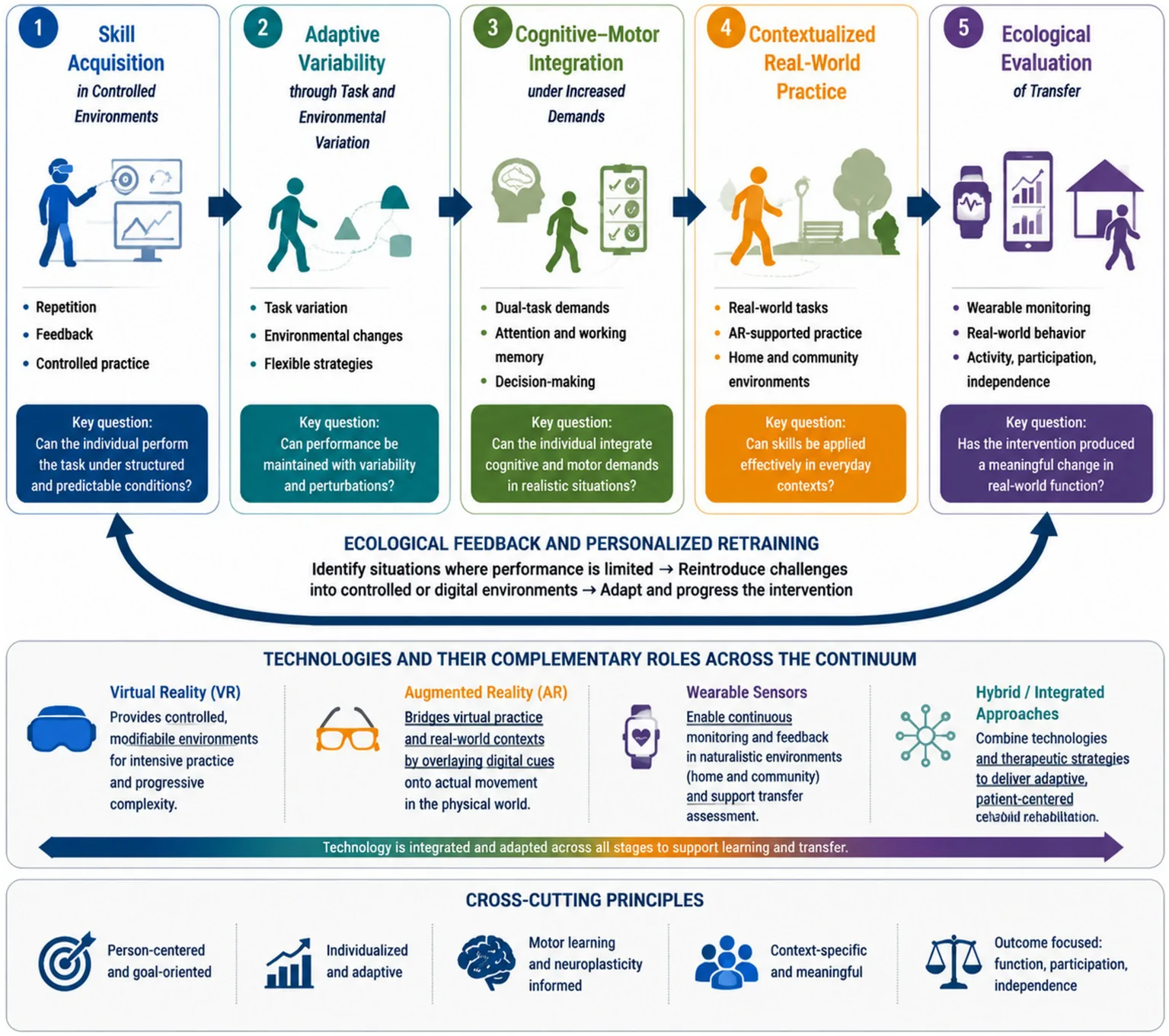
A transfer-oriented adaptive framework for digital neurorehabilitation.

## 6 Translating the framework into technology-enabled rehabilitation

Digital technologies should be viewed as complementary tools that support different stages of the transfer process rather than as independent rehabilitation solutions. The effectiveness of VR, AR, wearable sensing, and hybrid rehabilitation models depends not only on their ability to deliver intensive training but also on how effectively they facilitate progression from controlled skill acquisition toward meaningful real-world function. Within this transfer-oriented framework, VR may provide controlled environments for repeated practice, feedback, and progressive manipulation of task demands (45). AR may support the transition from digital practice to real-world activity by integrating digital cues with actual movement in meaningful physical contexts (42). Wearable technologies may extend assessment and feedback beyond clinical settings by capturing real-world behavior and identifying whether improvements achieved during training are reflected in daily performance (46, 47).

The greatest potential may therefore come from integrated and adaptive approaches that combine digital training, ecological monitoring, and individualized rehabilitation strategies. Rather than replacing conventional rehabilitation, technology should extend therapeutic opportunities by allowing continuous assessment, personalization, and progression across contexts. Future developments should focus not on creating increasingly complex digital environments alone, but on designing technology-supported interventions that promote generalization, long-term retention, and meaningful improvements in everyday function.

## 7 Future research priorities

Several limitations should be considered when interpreting this review. Narrative source selection may have introduced selection bias, while heterogeneity in neurological populations, interventions, and outcome measures limits comparisons across studies. Evidence on real-world transfer was sometimes indirect, restricting conclusions about generalization beyond trained tasks. Moreover, the proposed framework remains conceptual and unvalidated. Future research should directly evaluate transfer to untrained activities and real-world function using outcome frameworks that distinguish trained-task performance, near and far transfer, functional outcomes, and daily-life activity. Studies should investigate factors influencing transfer, including practice variability, cognitive-motor demands, sensory characteristics, and integration of digital and real-world practice. Combining clinical assessments with wearable and mobile monitoring may clarify whether improvements in functional capacity translate into meaningful changes in daily performance. Future interventions should also account for individual and environmental factors and investigate whether adaptive approaches improve generalization. Finally, the proposed framework should be empirically evaluated for its feasibility, clinical utility, and effectiveness in promoting real-world transfer.

## 8 Conclusion

Digital neurorehabilitation can improve motor learning and selected functional outcomes, but the extent to which these benefits generalize to everyday activities varies. The proposed transfer-oriented framework integrates adaptive practice, cognitive-motor demands, real-world application, and ecological evaluation to guide intervention design and assessment. Future research should directly evaluate transfer across functional outcomes and naturalistic environments and establish the feasibility and effectiveness of the framework.
